# Total reflection X‐ray fluorescence analysis of elemental composition of herbal infusions and teas

**DOI:** 10.1002/jsfa.10463

**Published:** 2020-05-26

**Authors:** Aleksandra Winkler, Mirjam Rauwolf, Johannes H Sterba, Peter Wobrauschek, Christina Streli, Anna Turyanskaya

**Affiliations:** ^1^ Atominstitut, TU Wien Vienna Austria

**Keywords:** herbs, teas, infusions, total reflection X‐ray fluorescence analysis, elemental composition, principal component analysis

## Abstract

**BACKGROUND:**

The elemental composition of herbal infusions and teas has not been sufficiently investigated. It could potentially be used for defining fingerprints for individual herbal / tea infusions, differentiation of botanical families, detecting the influence of packaging, and other purposes. The objective of this study was to determine the elemental composition, including the trace element content, of various herbal infusions and teas by means of total reflection X‐ray fluorescence analysis (TXRF), with a chemometrics approach using principal component analysis (PCA).

**RESULTS:**

This study determined the elemental composition of various herbal infusions and teas, including trace elements, using total reflection X‐ray fluorescence (TXRF). The methodology for the sample preparation was established, including the multiple‐steepings procedure for the two tea samples (Oolong and Pu‐erh). Data from 29 samples were collected. We hypothesized that the elemental content of infusions could reflect certain features, such as the influence of processing and the type of tea.

**CONCLUSION:**

A chemometric approach (PCA) was applied, and differences between teas and herbal infusions were found. This was further corroborated by explicit differentiation of one botanical family, Theaceae. The influence of packaging (tea bags) on herbal material was identified. The three types of tea (*Camellia sinensis*) appeared to be separated with PCA, and elemental concentrations in Pu‐erh changed with multiple steepings.

## INTRODUCTION

Herbal teas and infusions are popular and widely used. They have been used as herbal medicines since ancient times and their efficacy has been extensively corroborated. They can promote health and increase lifespan and enhance quality of life,^1^ and they display a broad spectrum of pharmacological activities, including antimicrobial, anti‐inflammatory, adaptogenic, stimulatory, and sedative action. Herbs and phytopreparations are effective in therapy and in the prevention of various diseases, e.g. cardiovascular and nervous, gastrointestinal disorders, dermatological conditions, and even different malignancies.^1^, ^2^

Herbal material contains multiple active compounds but, precisely because of their complex composition, herbs are challenging to analyze. There is a wealth of experience and expertise in organic compounds from plant material, including an array of established methods of qualitative and quantitative analysis. However, at the same time, the mineral composition of plants has been downplayed or underinvestigated.

There are renowned examples where particular elements have drawn the attention of scientists, such as manganese in tea leaves, selenium in garlic, or iron in spinach – usually due to supposed nutritional benefits. Potentially hazardous, toxic elements, e.g. lead, cadmium, mercury, arsenic, or the rising concerns about fluoride in tea^3^, ^4^ are controlled; however, even this aspect is scarcely investigated. Due to the lack of research, the World Health Organization states that it is currently unable to recommend limits for contaminants and residues in herbal materials and medicines and that it would be desirable to harmonize limits for toxic metals and standards.^5^, ^6^

Fortunately, in recent decades the development of cutting‐edge methods of elemental analysis have allowed comprehensive determination of the elements within raw and processed herbal material. Such investigations can focus on food authenticity, traceability, and the determination of geographical and botanical origin. The spectrometric methods employed often include variants of atomic absorption spectroscopy, such as flame atomic absorption spectrometry (FAAS) and similar techniques; inductively coupled plasma (ICP) techniques, e.g. optical emission spectrometry (ICP‐OES), formerly referred to as atomic emission spectroscopy (ICP‐AES), or mass spectrometry (ICP‐MS); and total reflection X‐ray fluorescence spectrometry (TXRF). Flame atomic absorption spectrometry is useful in the analysis of major and minor elements but is rather time consuming and to extend the analyzable range of elements it is often used in combination with other analytical techniques – e.g. FAAS with flame atomic emission spectrometry,^7^ FAAS with ICP‐MS,^8^ FAAS with ICP‐AES/ICP‐OES.^9^, ^10^ Inductively coupled plasma‐based techniques allow fast and accurate analysis of a wide range of elements but require matrix‐matching standards for quantification.^8^, ^11^, ^12^, ^13^, ^14^

Total reflection X‐ray fluorescence spectrometry offers a compelling alternative to the techniques mentioned above – a universal and inexpensive multi‐element method suitable for macro‐, micro‐ and trace element analysis, without the need for complex sample preparation.^15^ Detailed information on the method can be found in Klockenkämper and von Bohlen (2014).^16^ Total reflection X‐ray fluorescence was successfully applied for the analysis of teas^17^, ^18^, ^19^ and manufactured herbal blends.^18^ In the current work, we present our routine for TXRF analysis and show its application for the direct analysis of brewed teas and infusions of medical plants, which are often used for preparation of home‐made remedies and manufactured tea / herbal blends. The paper is based on the A. Winkler’s master's thesis: ‘Total reflection X‐ray fluorescence analysis of trace elements in black teas and herbal infusions’.^20^

In this study, we hypothesized that the elemental content of infusions of individual plant material can reflect certain features, i.e. affiliation of plant with a botanical family, influence of production processing (i.e. loose herbs or tea bags), and, specifically for teas (*Camellia sinensis*), the type of tea and influence of sample preparation when the multiple steeping procedure is used. The objective of the study was to apply multi‐elemental TXRF analysis to the infusions of selected individual herbs and teas, and further evaluate them by chemometrics analysis (PCA), to find specific fingerprints or common features within the analyzed samples based on their elemental composition.

## MATERIALS AND METHODS

### Sample selection

Thirteen kinds of plants and three different teas were selected. Herbal samples included anise, calamus, chamomile, fennel, lapacho (Pau d’Arco), lavender, mallow, nettle, peppermint, sage, thyme, valerian, and yarrow. The selection was made subjectively; priority was given to herbs that are well known as traditional medicines or home remedies. For example, anise, calamus, chamomile, fennel, mallow, peppermint, and yarrow are used for relieving stomach conditions; sage and thyme as cough relief; valerian as a sedative, and lapacho, lavender and nettle have a wide range of pharmacological effects and are used in a number of conditions. All the herbs are often used as components in manufactured teas and tea‐like herbal blends. We focused on investigating the single herbs, to test whether we could determine traits specific to each sample.

Most of the loose herbal samples were acquired from Austrian pharmacies or supermarkets, except for the fennel 1 sample, which was purchased in a Latvian pharmacy, and one of the peppermint samples (wild peppermint – peppermint W), which was hand‐picked in Latvia (Pļaviņas region). Herbs in tea bags were bought in Austrian supermarkets. Most herbal samples are typical for the flora of Europe, with the exception of lapacho, a tree that is common from north‐western Mexico to north‐western Argentina.

The teas used were Darjeeling, Oolong and Pu‐erh. Depending on the processing procedures used, teas can be divided into green tea (non‐fermented), oolong tea (semi‐fermented), black tea (fully fermented by oxidizing enzymes) and dark tea (post‐fermented by microbes).^21^ Our project therefore covered three of those groups – oolong tea, black tea (Darjeeling), and dark tea (Pu‐erh). Samples of Darjeeling were purchased in Austrian supermarkets. Samples of Oolong and Pu‐erh were bought in China. For these types of tea, several steepings are recommended (Oolong: three to four times; Pu‐erh tea: up to 12 times). We therefore analyzed extracts from consecutive brewing steps.

Table 1 provides a complete list of herbal material and teas analyzed in this study.

### TXRF spectrometer

The Atomika 8030C TXRF(Atomika Instruments GmbH, Oberschleissheim, Germany) spectrometer was employed for the measurements. The spectrometer performs simultaneous and fast determination of all elements within the range from sodium to uranium and provides detection limits down to the μg/L level. The spectrometer has a 2.5 kW high power X‐ray tube with a molybdenum‐tungsten (Mo/W) mix anode. An 80 mm^2^ Si(Li) detector was used for the analysis of the characteristic fluorescence radiation. The measurement parameters were: measurement time (live time, LT) 1000 s; excitation Mo‐Kα; filter 20 μm Zr foil; tube current 37 mA. Measurements were performed in air.

### Sample carrier cleaning

The Atomika 8030C spectrometer is designed for sample carriers that are 3 cm in diameter and about 3 mm in height. Usually quartz reflectors are used. After a thorough cleaning, which included ultrasonic baths with Decon 90 cleaning solution (about 20 mL Decon 90 in 300 mL tri‐distilled water) and nitric acid (20%–30% HNO_3_), each reflector was measured for 1000 s. When no contamination was detected (see supplementary material) the carriers were used for sample measurements.

The beakers used for sample preparation were cleaned in a similar way to sample carriers, and controlled before every use. In this case, about 150 mL of tri‐distilled water was boiled in the cleaned beaker for a few minutes; 10 μL of this water was pipetted on a clean quartz reflector, tried and measured for 1000 s. Only if this procedure did not reveal any contamination was the beaker used for sample preparation.

More detailed information on the cleaning procedure can be found in Winkler (2017).^20^

### Sample preparation

The samples selected for analysis are often used in tea / herbal blends but if infusions of such a blended mix were to have been measured, the contribution of single component could not have been determined. In our project we exclusively aimed to detect the elemental composition of each extract separately; the established sample preparation protocol was therefore identical for all the loose herbs, and for the Darjeeling tea samples.

Each portion of plant material was weighed using an analytical balance; 50 mL of boiling hot tri‐distilled water was poured over 1 g of the material (i.e. leaves, bark, flowers, fruits), which then was steeped in the hot water for 10 min. This procedure was chosen to maximize the resemblance to ‘homemade’ tea.

As the quantity of the herb in a tea bag was slightly different for each product, 150 mL of boiling hot tri‐distilled water was used for each tea bag. The tea bag was then left in the hot water for 10 min. Tea bags ranged from 1.5 g to 2.25 g in mass.

As mentioned above, Oolong and Pu‐erh teas require multiple steepings, due to the specific preparation recommendations for these types of tea. The same leaves were brewed several times using fresh water for the preparation of each steeping. The infusions from each brewing step were taken as separate samples.

For the preparation of the Pu‐erh tea samples, the whole package (a small disc of pressed tea leaves weighting about 4.96 g) was used. These leaves were steeped five times in 85 mL of tri‐distilled water. Each time, leaves were left to brew for 30 s. Then the liquid infusion (sample) was filled in a test tube. Fresh boiling water was added to the leaves and the next sample was taken after 30 s.

For the Oolong tea samples, 1 g of the tea leaves was steeped twice in 50 mL of tri‐distilled water. Each steeping took 10 min.

Finally, after an infusion was ready and cooled down, approximately 500 mg of pure sample were spiked with a suitable amount of Ga to give a final Ga concentration of 1 mg/L (the exact amounts were controlled using laboratory scales, and correction factors were applied to calculate the final concentrations when needed). On five different quartz sample carriers, an aliquot of 10 μL of Ga‐spiked specimen were pipetted. Samples were dried on a heating plate (5 min) and then measured for 1000 s live time.

### Quantification and data analysis

Quantification of the samples was performed with respect to the internal standard using the software of the spectrometer. To validate the built‐in calibration of the software a standard reference material (NIST SRM 1640 – Natural Water) was used (additional information in supplementary material). Mean concentrations and standard deviations (out of five replicates) for every element in each sample were calculated with Microsoft Excel. Principal component analysis (PCA) was performed using R statistical software. For this purpose, the data were normalized using standard deviation. Only the elements (i) which were present in all the samples investigated and (ii) for which the data of all five measurements were available were included in the PCA.

## RESULTS AND DISCUSSION

**Figure 1:**
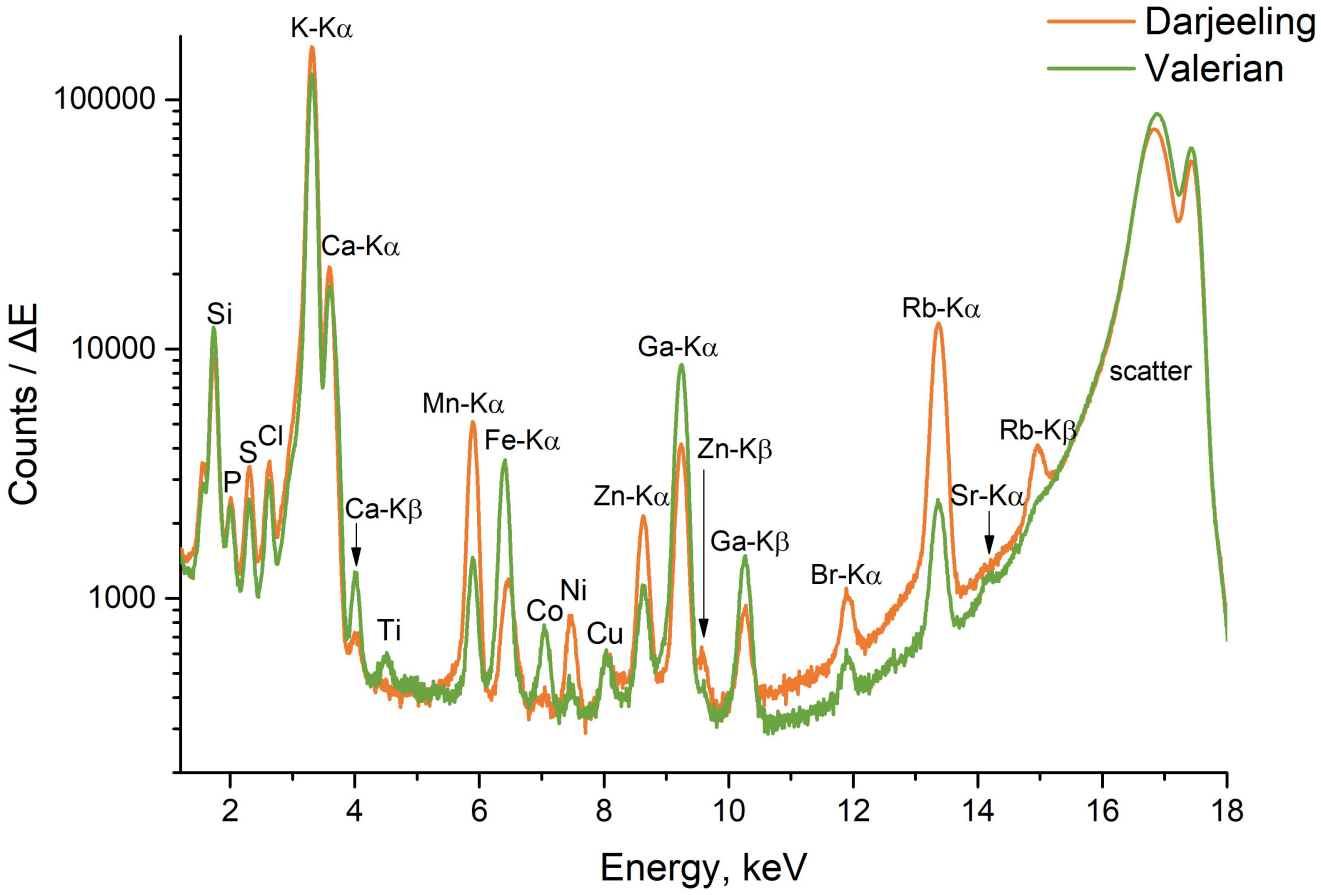
Spectra of typical tea (Darjeeling sample) and typical herbal infusion (Valerian 1 sample).

The analysis of the 29 different herbal infusions and tea samples identified 13 ‘default’ elements, which were found in most of the measured samples: P, S, Cl, K, Ca, Mn, Fe, Ni, Cu, Zn, Br, Rb, and Sr. There are also three ‘occasional’ elements, which could be found only in few samples: Sc, Co, and Ba. The acquired element concentrations for all the samples are presented in the Table 2 (default elements) and Table 3 (occasional elements). They are given as the mean values out of the five replicated measurements and their standard deviations, unless stated otherwise. For reference only we have also included the results for elements present in four measurements out of five in Tables 2 and 3. For this reason, Ti is not included in the list of occasional elements, although it was detected in two and three measurements out of five for valerian 1 and 2 correspondingly (see the Ti peak in the spectrum on Fig. 1). Scandium is difficult to confirm in the presence of calcium, due to the spectral overlap of Sc‐Kα with Ca‐Kβ, in the case of nettle; Sc was determined by the fitting software of the Atomika 8030C spectrometer in four out of five samples.

**Table 1:** List of all the analyzed samples

	Nr.	Sample name	Latin name	Part of the plant	Source
Herbs	1	Anis	*Pimpinella anisum* L.	Fruits	Purchased in Austria
	2	Calamus	*Acorus calamus* L.	Rhizome	Purchased in Austria
	3	Chamomile	*Matricaria recutita* L.	Flowers	Purchased in Austria
	4	Chamomile TB			Egypt, East Europe (purchased in Austria)
	5	Fennel 1	Foeniculum vulgare Mill.	Fruits	Purchased in Latvia
	6	Fennel 2			Purchased in Austria
	7	Fennel TB			Purchased in Austria
	8	Lapacho	*Handroanthus impetiginosus*	Bark	Purchased in Austria
	9	Lavender	Lavandula angustifolia Mill.	Flowers	Purchased in Austria
	10	Mallow	*Malva silvestris* L. *and Malva neglecta* L.	Leaves	Purchased in Austria
	11	Nettle	*Urtica dioica* L.	Leaves	Romania (purchased in Austria)
	12	Peppermint	*Mentha piperita* L.	Leaves	Purchased in Austria
	13	Peppermint W			collected in Latvia, Pļaviņas region
	14	Peppermint TB			Egypt, East Europe (purchased in Austria)
	15	Sage	*Salvia officinalis* L.	Leaves	Purchased in Austria
	16	Sage TB			Purchased in Austria
	17	Thyme	*Thymus vulgaris* L.	Whole plant	Egypt, Morocco, East Europe, Turkey (purchased in Austria)
	18	Valerian 1	*Valeriana officinalis* L.	Roots	Purchased in Austria
	19	Valerian 2			
	20	Yarrow	*Achillea millefolium* L.	Whole plant	Purchased in Austria
Tea	21	Darjeeling	*Camellia sinensis (*L.*) Kuntze*	Leaves	Darjeeling region, North India (purchased in Austria)
	22	Darjeeling Bio			Darjeeling region, North India (purchased in Austria)
	23–24	Oolong (Oolong#1–#2)			China, purchased in Yunnan region
	25–29	Pu‐erh (Pu‐erh#1–#5)			China, purchased in Yunnan region

For herbs, if not stated otherwise, loose material was used; plant material received from several sources is differentiated by index number. TB stands for manufactured tea bags. The sample obtained from hand‐picked wild peppermint leaves is denoted as Peppermint W. Several steepings of Oolong and Pu‐erh teas are denoted with # and index number. For tea only loose material was used.

**Table 2:** Means and standard deviations of the default elements for all measured samples are given in mg/L

	P‐K	S‐K	Cl‐K	K–K	Ca‐K	Mn‐K	Fe‐K	Ni‐K	Cu‐K	Zn‐K	Br‐K	Rb‐K	Sr‐K
**Herbs**													
Anis	6.22 ± 0.32	13.9 ± 0.6	18.7 ± 0.7	233 ± 5	35.7 ± 0.5	0.095 ± 0.005	0.18 ± 0.04	0.0570 ± 0.0024	0.0357 ± 0.0025	0.1130 ± 0.0032	0.101 ± 0.004	0.1351 ± 0.0026	0.0622 ± 0.0017
Calamus	27 ± 6	18 ± 4	28 ± 5	135 ± 13	7.3 ± 0.7	0.231 ± 0.016	0.38 ± 0.05	—	0.0393 ± 0.0031	0.051 ± 0.006	0.0579 ± 0.0016	0.0806 ± 0.0031	0.0171 ± 0.0015
Chamomile	55.5 ± 2.8	37.7 ± 2.2	117 ± 9	456 ± 22	29.7 ± 1.0	1.44 ± 0.04	0.081 ± 0.024	0.1260 ± 0.0032	0.112 ± 0.006	0.308 ± 0.012	0.616 ± 0.022	0.142 ± 0.005	0.0235 ± 0.0013
Chamomile TB	18.9 ± 1.6	31.4 ± 1.2	197 ± 20	277.7 ± 3.1	42.6 ± 0.4	0.322 ± 0.009	0.105 ± 0.025	0.0312 ± 0.0029	0.0679 ± 0.0026	1.051 ± 0.006	0.358 ± 0.006	0.0535 ± 0.0026	0.4317 ± 0.0023
Fennel 1	5.7 ± 0.8	37.9 ± 2.0	208 ± 13	550 ± 40	92.5 ± 2.3	0.189 ± 0.005	0.32 ± 0.10	0.021 ± 0.005	0.0833 ± 0.0025	0.877 ± 0.009	0.298 ± 0.006	0.365 ± 0.007	0.739 ± 0.005
Fennel 2	5.2 ± 0.4	6.23 ± 0.30	13.0 ± 0.6	113 ± 4	11.24 ± 0.23	0.0126 ± 0.0021	0.036 ± 0.021	—	0.0188 ± 0.0028	0.020 ± 0.007	0.0333 ± 0.0014	0.0210 ± 0.0016	0.0209 ± 0.0015
Fennel TB	8.3 ± 1.1	25.6 ± 0.7	99.5 ± 3.3	347 ± 8	24.98 ± 0.30	0.097 ± 0.006	0.234 ± 0.029	0.0300 ± 0.0024	0.1163 ± 0.0034	0.219 ± 0.004	0.060 ± 0.005	0.1205 ± 0.0027	0.2461 ± 0.0014
Lapacho	—	3.8 ± 0.4	5.2 ± 1.8	39.1 ± 0.8	16.6 ± 0.4	0.110 ± 0.005	0.127 ± 0.028	0.0106 ± 0.0028	0.0158 ± 0.0021	0.128 ± 0.021	0.190 ± 0.008	0.0596 ± 0.0022	0.1559 ± 0.0031
Lavender	12.3 ± 1.2	18.4 ± 1.1	33.7 ± 1.6	319 ± 7	37.5 ± 0.9	0.193 ± 0.009	0.177 ± 0.022	0.0143 ± 0.0025	0.089 ± 0.004	0.1294 ± 0.0030	0.0943 ± 0.0033	0.321 ± 0.005	0.0652 ± 0.0020
Mallow	113 ± 10	99.2 ± 2.8	122.0 ± 3.3	1001 ± 35	261 ± 5	0.46 ± 0.04	0.43 ± 0.07	0.108 ± 0.011	0.191 ± 0.016	0.58 ± 0.04	0.220 ± 0.007	0.123 ± 0.007	0.210 ± 0.005
Nettle	15.2 ± 1.7	76 ± 8	59 ± 4	710 ± 60	176.5 ± 2.1	0.178 ± 0.007	0.21 ± 0.04	0.025 ± 0.008	0.068 ± 0.007	0.460 ± 0.025	0.079 ± 0.005	0.083 ± 0.009	0.951 ± 0.027
Peppermint	54.7 ± 1.9	36.3 ± 1.0	269 ± 11	793 ± 18	101.6 ± 1.1	0.349 ± 0.005	0.080 ± 0.011	0.053 ± 0.004	0.099 ± 0.008	0.182 ± 0.010	0.389 ± 0.010	0.088 ± 0.004	0.0627 ± 0.0028
Peppermint W	16.2 ± 1.3	65.8 ± 0.5	138.1 ± 1.6	284.9 ± 2.2	55.76 ± 0.19	0.289 ± 0.004	0.16 ± 0.05	0.0172 ± 0.0034	0.0394 ± 0.0032	0.116 ± 0.006	0.189 ± 0.004	0.089 ± 0.004	0.427 ± 0.005
Peppermint TB	22.4 ± 1.7	108 ± 6	61.9 ± 2.3	123 ± 4	132 ± 4	1.139 ± 0.020	0.269 ± 0.008	0.016 ± 0.004	0.050 ± 0.008	0.136 ± 0.008	0.974 ± 0.021	0.1903 ± 0.0035	0.380 ± 0.010
Sage	7.9 ± 1.2	39.3 ± 1.1	28.4 ± 1.6	306 ± 10	113.5 ± 1.6	0.257 ± 0.005	0.071 ± 0.012	0.0120 ± 0.0028	0.0395 ± 0.0024	0.176 ± 0.005	0.114 ± 0.004	0.274 ± 0.011	0.0657 ± 0.0022
Sage TB	10.7 ± 1.2	22.3 ± 0.8	20.1 ± 2.6	235 ± 7	78.6 ± 1.0	0.295 ± 0.007	0.25 ± 0.04	0.0229 ± 0.0030	0.021 ± 0.005	0.1582 ± 0.0022	0.133 ± 0.005	0.166 ± 0.006	0.0909 ± 0.0032
Thyme	15.4 ± 2.0	25.8 ± 0.6	42.3 ± 1.2	273.4 ± 2.9	123.4 ± 1.4	0.858 ± 0.018	0.24 ± 0.04	0.0135 ± 0.0029	0.062 ± 0.004	0.1782 ± 0.0032	0.298 ± 0.006	0.494 ± 0.010	0.0798 ± 0.0029
Valerian 1	33.6 ± 2.6	17.6 ± 1.4	10.2 ± 0.7	257 ± 9	5.17 ± 0.20	0.284 ± 0.009	0.47 ± 0.04	0.0221 ± 0.0031	0.025 ± 0.004	0.128 ± 0.006	0.0114 ± 0.0009	0.101 ± 0.004	0.0167 ± 0.0022
Valerian 2	23.6 ± 1.0	15.8 ± 0.5	8.48 ± 0.23	209.3 ± 2.6	6.97 ± 0.07	0.345 ± 0.004	0.80 ± 0.11	0.0204 ± 0.0026	0.0406 ± 0.0016	0.112 ± 0.004	0.0163 ± 0.0017	0.1498 ± 0.0016	0.0113 ± 0.0018
Yarrow	41.7 ± 1.4	19.1 ± 0.6	126.2 ± 2.2	457 ± 7	28.21 ± 0.14	0.381 ± 0.010	0.18 ± 0.07	0.056 ± 0.009	0.0691 ± 0.0034	0.117 ± 0.007	0.828 ± 0.014	0.142 ± 0.004	0.0825 ± 0.0019
**Teas**													
Darjeeling	46.8 ± 1.5	42.8 ± 0.7	18.2 ± 0.7	525 ± 7	4.69 ± 0.15	3.267 ± 0.022	0.166 ± 0.011	0.203 ± 0.005	0.078 ± 0.005	0.542 ± 0.004	0.0918 ± 0.0027	2.006 ± 0.019	0.015 ± 0.004^a^
Darjeeling Bio	33.1 ± 2.4	34.6 ± 1.6	13.8 ± 0.8	495 ± 12	3.50 ± 0.15	4.29 ± 0.06	0.075 ± 0.027	0.171 ± 0.006	0.1269 ± 0.0023	0.433 ± 0.015	0.0646 ± 0.0024	1.403 ± 0.015	0.0143 ± 0.0029
Oolong#1	26.0 ± 1.0	13.40 ± 0.23	27.4 ± 0.4	286.3 ± 2.6	1.22 ± 0.04	2.035 ± 0.011	0.021 ± 0.007	0.0293 ± 0.0019	0.13 ± 0.23	0.196 ± 0.009	0.0103 ± 0.0013	0.5605 ± 0.0025	—
Oolong#2	7.0 ± 0.8	4.3 ± 0.5	3.2 ± 0.6	92.0 ± 3.4	0.419 ± 0.021	0.600 ± 0.010	0.020 ± 0.006	—	0.0104 ± 0.0017	0.057 ± 0.004	—	0.1729 ± 0.0029	—
Pu‐erh#1	10.26 ± 0.13	4.48 ± 0.32	3.98 ± 0.28	62.4 ± 1.0	0.724 ± 0.014	0.489 ± 0.011	0.074 ± 0.006	0.0131 ± 0.0031	—	0.037 ± 0.004	0.0080 ± 0.0018^a^	0.317 ± 0.006	0.0092 ± 0.0013
Pu‐erh#2	38.5 ± 0.7	17.96 ± 0.31	12.9 ± 0.4	273.9 ± 3.3	2.91 ± 0.11	2.416 ± 0.025	0.32 ± 0.04	0.0612 ± 0.0031	0.0244 ± 0.0014	0.1338 ± 0.0031	0.0374 ± 0.0017	1.430 ± 0.024	0.0479 ± 0.0026
Pu‐erh#3	50.6 ± 3.0	24.2 ± 1.0	15.2 ± 1.2	372 ± 12	4.93 ± 0.08	3.75 ± 0.09	0.603 ± 0.020	0.084 ± 0.005	0.0368 ± 0.0020	0.198 ± 0.008	0.0494 ± 0.0034	1.96 ± 0.05	0.0694 ± 0.0018
Pu‐erh#4	26.6 ± 1.8	12.3 ± 0.4	5.6 ± 0.9	229.4 ± 3.3	2.97 ± 0.08	2.223 ± 0.029	0.265 ± 0.021	0.049 ± 0.004	0.0245 ± 0.0026	0.116 ± 0.006	0.0244 ± 0.0017	1.190 ± 0.011	0.0339 ± 0.0021
Pu‐erh#5	8.9 ± 1.4	4.26 ± 0.18	2.20 ± 0.25	111.7 ± 3.5	1.71 ± 0.06	0.955 ± 0.018	0.18 ± 0.05	0.0186 ± 0.0015	0.0122 ± 0.0008	0.0586 ± 0.0017	0.0098 ± 0.0009	0.591 ± 0.015	0.0122 ± 0.0014^a^

Means and standard deviation were calculated from the results of five measurements for each sample, with the exception of the fourth steeping of the Pu‐erh tea (Pu‐ehr#4), which was measured six times.

a Detected in four out of five measurements.

**Table 3:** Means and standard deviations of the occasional elements for all measured samples are given in mg/L

	Sc‐K	Co‐K	Ba‐L
**Herbs**			
Anis	‐	0.012±0.004	‐
Calamus	‐	‐	‐
Chamomile	‐	0.016±0.005	‐
Chamomile TB	‐	0.0199±0.0019	‐
Fennel 1	‐	0.035±0.006	‐
Fennel 2	‐	‐	‐
Fennel TB	‐	‐	‐
Lapacho	‐	‐	0.210±0.035
Lavender	‐	‐	‐
Mallow	‐	0.055±0.011	‐
Nettle	0.24±0.05^a^	0.030±0.008	‐
Peppermint	‐	0.039±0.010	‐
Peppermint W	‐	‐	0.165±0.031
Peppermint TB	‐	‐	‐
Sage	‐	0.0146±0.0026	‐
Sage TB	‐	‐	‐
Thyme	‐	0.0165±0.0029^a^	0.204±0.020^a^
Valerian 1	‐	‐	‐
Valerian 2	‐	‐	‐
Yarrow	‐	0.0146±0.0035	‐
**Teas**			
Darjeeling	‐	‐	‐
Darjeeling Bio	‐	0.0161±0.0013^a^	‐
Oolong#1	‐	‐	‐
Oolong#2	‐	‐	‐
Pu‐erh#1	‐	‐	‐
Pu‐erh#2	‐	‐	‐
Pu‐erh#3	‐	0.0125±0.0017^a^	‐
Pu‐erh#4	‐	‐	‐
Pu‐erh#5	‐	‐	‐

Means and standard deviation were calculated from the results of five measurements for each sample, with the exception of the fourth steeping of the Pu‐erh tea (Pu‐ehr#4), which was measured six times.

a Detected in four out of five measurements.

As stated above, only the elements which were found in all five samples, and for which all five measurements were quantifiable, were included in the PCA.

### Elemental composition of infusions and teas

Figure 1 shows a comparison of the typical spectra from a tea and a herbal infusion. The mineral content of the tea has been well investigated,^17^, ^19^ and as expected, Mn and Rb concentrations in tea sample were higher, than in the herbal infusion.

**Figure 2:**
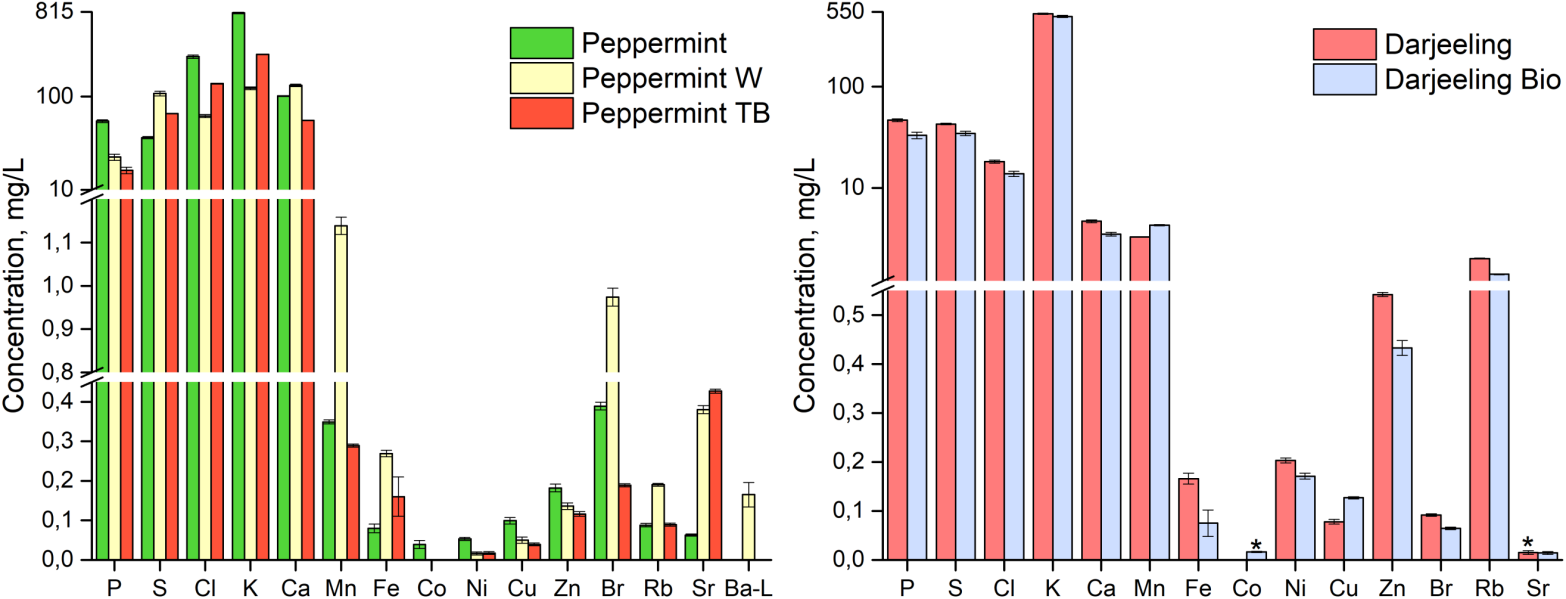
Comparison of the average values and standard deviations within similar plant material. Left‐hand side: Peppermint samples. Note the logarithmic scale in the range 10–815 mg/L. Right‐hand side: Darjeeling samples. Note the logarithmic scale in the range 1,2–550 mg/L. Asterisk marks the elements detected only in four out of five measurements.

We aimed to identify the potential fingerprints for individual herbal / tea infusions. Naturally, however, even the samples of the same plant material, but obtained from different sources, demonstrate variations in the concentrations of elements (Fig. 2). Factors such as plant origin, soil composition, production processing, and packaging can be responsible for these variations.

**Figure 3:**
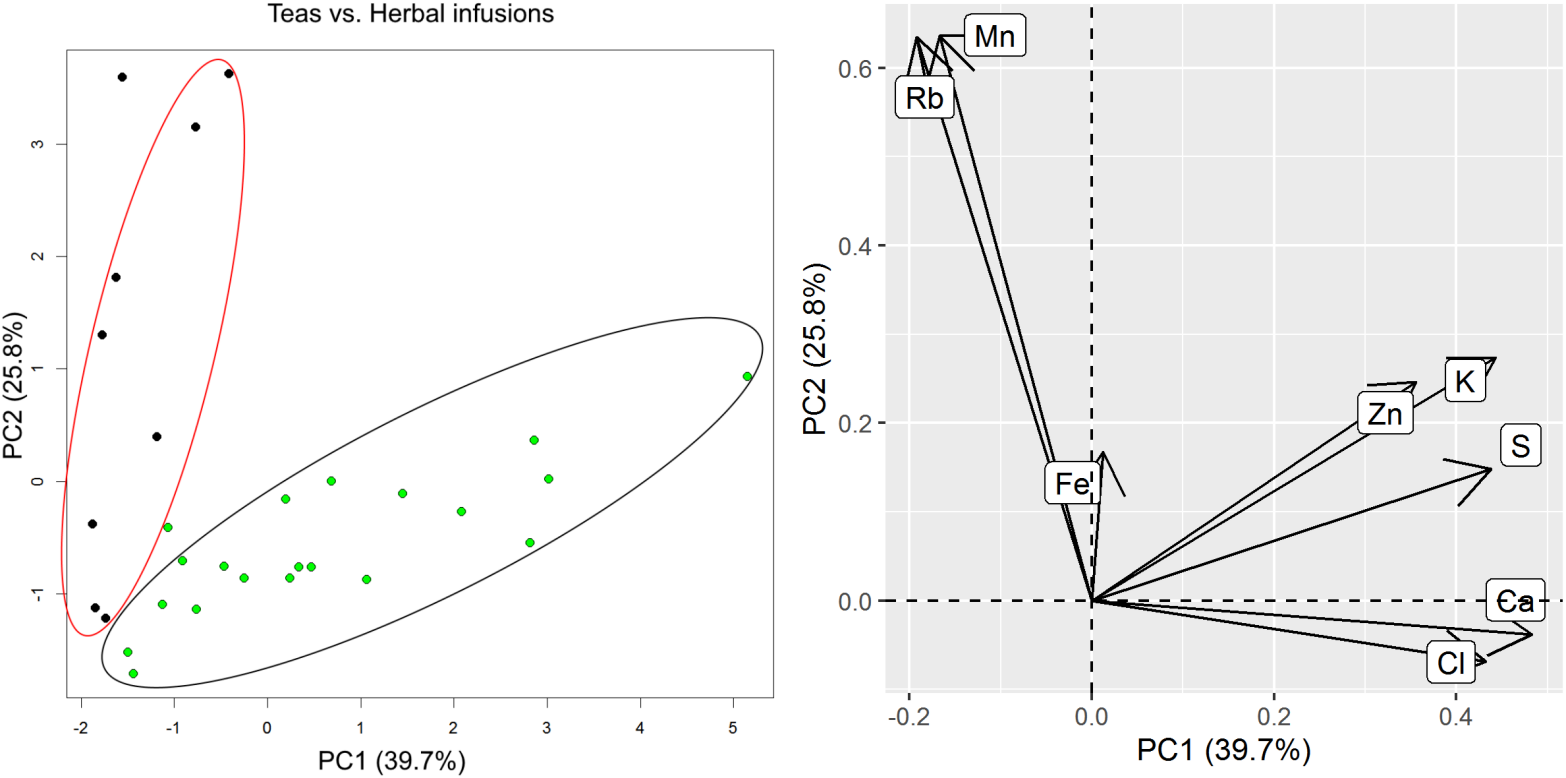
Teas versus herbal infusions: PCA plot (left) and variables factor map (right). On the PCA plot tea is represented by black dots and herbal infusions by green dots.

To evaluate the data trends, we therefore performed PCA, using the mineral content of the analyzed samples as chemical descriptors. Principal component analysis is normally used for data with large numbers of dimensions. The analysis serves to structure, simplify and illustrate the extensive data sets by approximating a large number of statistical variables by a smaller number of possible linear combinations (the so‐called main components). As we had the two apparent groups within our data set – herbal infusions and teas – these groups were compared in the first instance. The result can be seen on Fig. 3. PC1 and PC2 are the most meaningful components, representing 65.5% of the total variance (PC1 = 39.7%, PC2 = 25.8%), so they were chosen for graphical representation. Contributions of the elements to the first (*x*‐axis) and the second (*y*‐axis) PCA dimensions are shown on the right (Mn and Rb contribute the most in PC2).

The distinct separation of the herbal infusions and teas can be seen. We could demonstrate that the method of TXRF is sensitive enough to measure the relevant elements and covers wide range of elements – not only metals, but also non‐metallic elements (e.g. S, Cl). Broader range of elements might aid the differentiation of the samples. The method of sample preparation – infusions – is also practical and straightforward and was adequate for characterization of the samples. It is important to note that we did not detect lead (Pb) or other potentially hazardous and toxic metals (e.g. Cd, As, Hg) in any of our samples.

Similar studies, aiming to differentiate teas and herbs, were performed by Dalipi *et al*.^18^ and Veljković *et al*.^22^ Dalipi *et al*., and compared the manufactured mixed herbal tea bags and teas (various kinds of green, black, and oolong) using TXRF. In their case PCA was used to differentiate between green, black, and oolong teas, mixed herbs, and ginseng infusions, and revealed K, Ca, Ba, and Sr as the most significant variables for differentiation. Remarkably, Pb was detected in low concentration in the herbal infusions (0.25–0.50 mg/kg). Veljković *et al*. used ICP‐AES for analysis of teas (black and green) and other herbs (manufactured tea bags). The group of teas (compared with three herbal groups) could be distinguished by means of PCA based on higher concentrations of Cu, Mn, As, Al, and Pb.

These studies support our assumption, that various plant materials can be characterized and differentiated based on the elemental composition only. The difference in the relevant assigned elements can be explained by different types of analyzed herbal material (manufactured, mixed herbs, tea bags, single herbs) and its origin, different approaches to the sample preparation, various analytical methods employed, etc. The influence of sample preparation was investigated in numerous studies, which demonstrated that different elements have different extractability.^13^, ^23^

We believe that the clear separation obtained in our case can be explained by selected samples – only single herbs and no mixed samples, and the possible influence of the production processing or tea bag materials itself (see below) was minimal, as in our study only four types of herbs were packed in tea bags.

### Differentiation of botanical families

Chemotaxonomy is a branch of science aiming to classify biological organisms (plants) based on the ‘perceptible differences and similarities in their biochemical compositions’.^24^ It was demonstrated in a study by Arceusz *et al*. that it is possible to identify and classify, according to the plant families, the digested raw plant material based on elemental composition.^25^ The representatives of five plant families were investigated in their study, including Apiaceae, Asteraceae, and Lamiaceae, with regard to microelements (B, Zn, Fe, and Na) and macroelements (Mg, Ca, and K).

We hypothesized, that infusions of plants belonging to the same botanical family and sharing morphological similarities, can also display resemblance in mineral composition. We therefore grouped the plant material, independently of the formulation (loose or in tea bags; or steeping – for teas), according to its botanical families and, thus, identified four groups corresponding to the following families:

- Apiaceae: samples anise, fennel 1 and fennel 2;
- Asteraceae: chamomile, chamomile TB and yarrow;
- Lamiaceae: lavender, peppermint, peppermint W, peppermint TB, sage, sage TB and thyme;
- Theaceae: Darjeeling, Darjeeling bio, Oolong #1–#2, Pu‐erh #1–#5.

**Figure 4:**
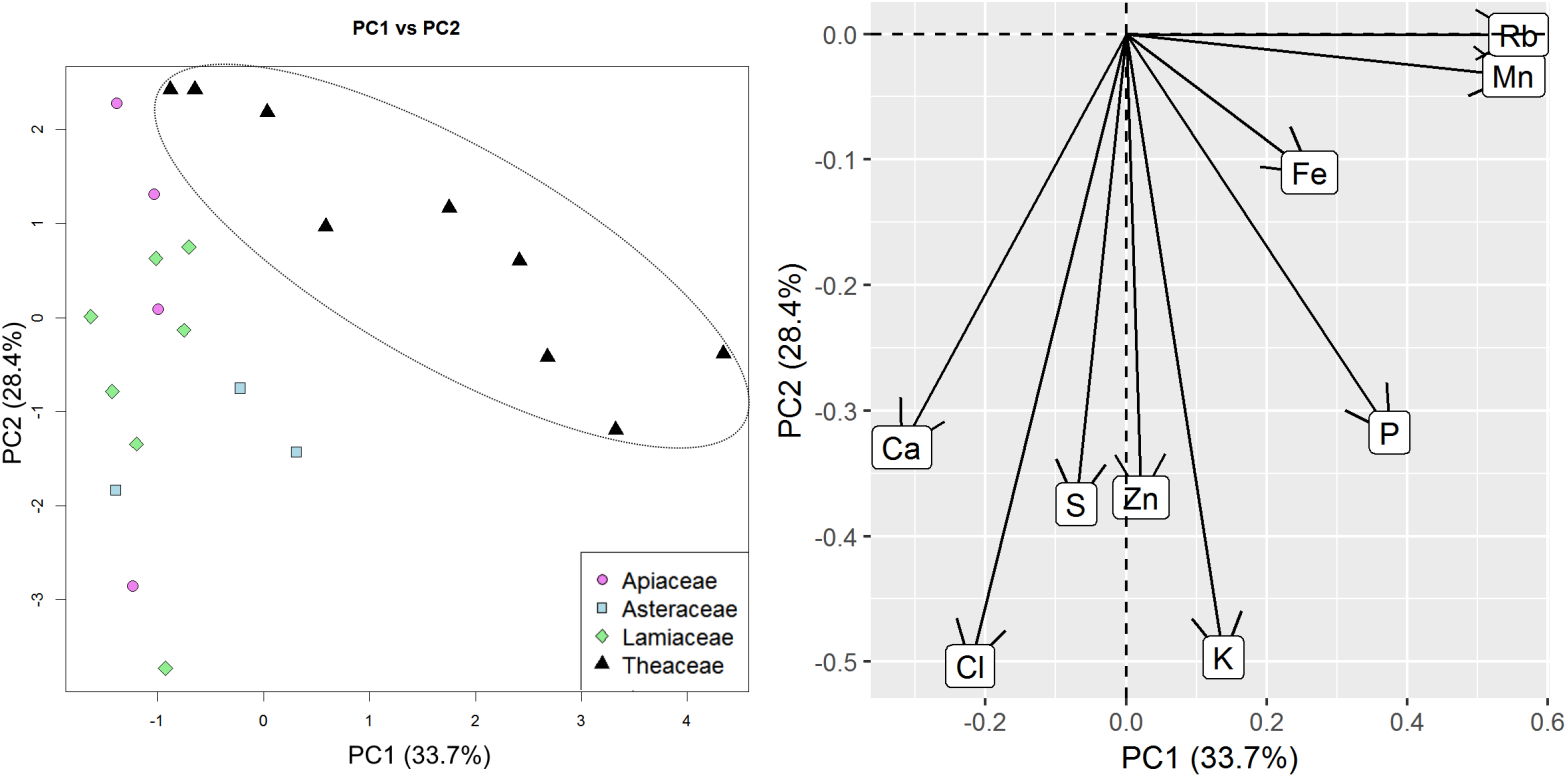
Analysis on the herbal families: PCA plot (left) and variables factor map (right).

The results are displayed in Fig. 4. It can be seen that the tea samples (Theaceae) can be distinguished as a separate group. As for the Apiaceae, Asteraceae, Lamiaceae families, no clear clustering can be observed.

The reasons for this could be the sample preparation and the differential extractability of various elements into infusions mentioned above, which would cause the individual traits of herbs to be obscured. The fact that within our analysis we combined samples, representing different parts of the plant – in case of Asteraceae (flowers, whole plant) and Lamiaceae (flowers, leaves, whole plant) – could also have an impact on the outcome. For example, De La Calle *et al*. performed a TXRF analysis of digested herbal material (various plants), represented by different anatomical parts, and clearly identified groups corresponding to flower, leaf or fruit using linear discriminant analysis – confirming the common traits in the elemental composition of those organs.^26^ However, Arceusz *et al*. used different parts of the plants as well, which did not hinder differentiation.^25^ Finally, it is likely, that the sample pool of our study is not sufficient, and analysis of more samples could help to reveal the trend for separation between the other three plant families.

### Influence of packaging

**Figure 5:**
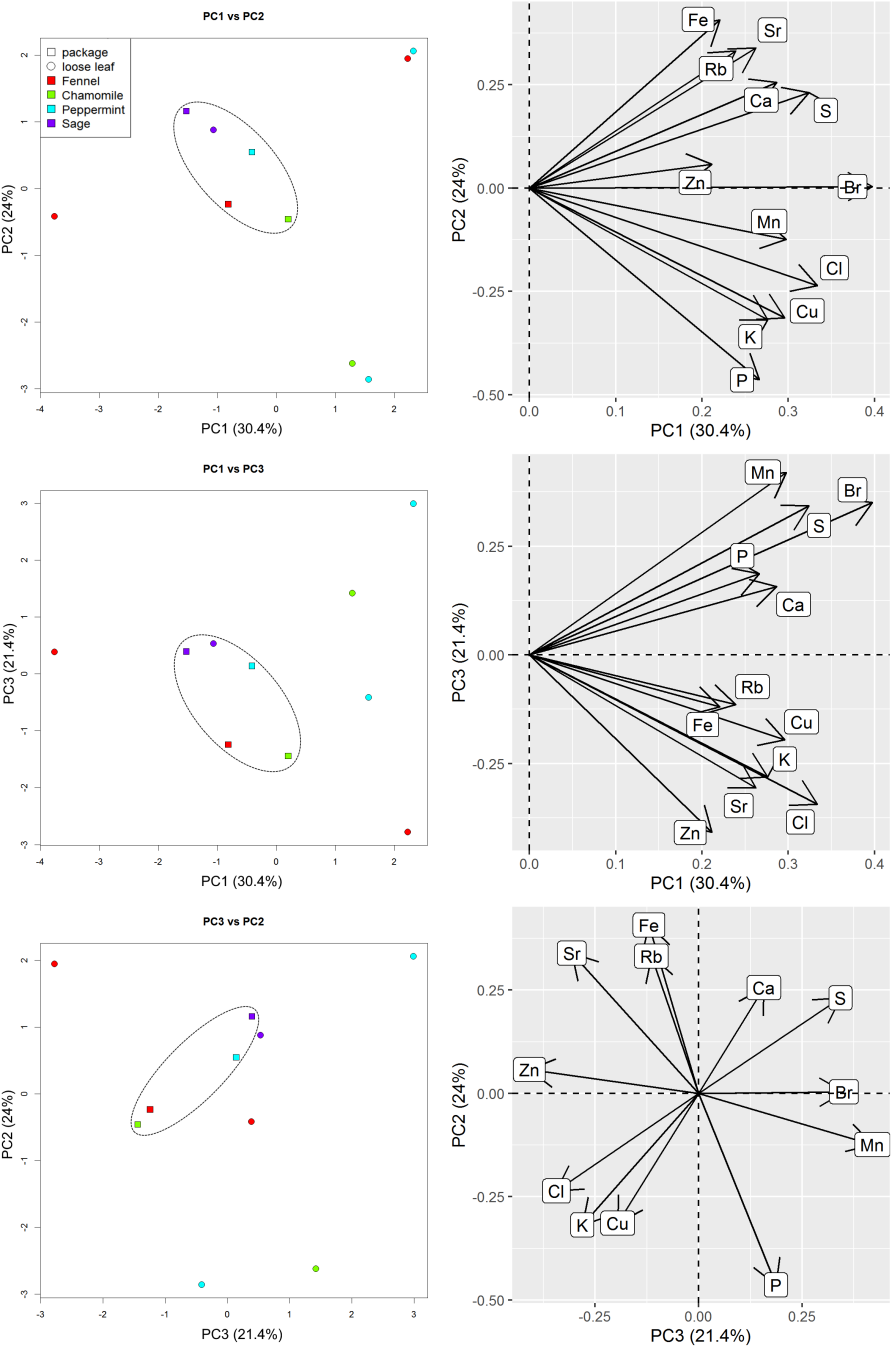
Loose herbal material and packaged herbal tea: PCA plot (left) and variables factor map (right).

Within our sample set, four herbs were present in both loose and packaged tea bag forms, namely chamomile, fennel, peppermint and sage. To estimate the possible influence of manufacturing / packaging on the extracts we also ran a PCA on this subset of the data. Figure 5 presents the three main components PC1, PC2, and PC3 for this comparison. In all three subfigures, loose herbal material samples have little in common; they are scattered over the whole plot, and are often rather far away from the corresponding packaged teas, except for the sage samples. At the same time, similarities in packaged teas are notable. Tea bags (marked as squares) are grouped in the center of the plots, independently of the herb. Therefore, this effect cannot be attributed to the type of herb, as the plants belong to the three different botanical families (Apiaceae, Asteraceae, Lamiaceae). Different organs of the plants were also present (flowers, fruits, leaves). It should be noted that plant material is crushed before packaging in the production of tea bags, so the extractability of the components will be increased in comparison with crude material. Furthermore, some unwanted elemental contamination might happen during production. Finally, the elemental composition of the tea bag material itself deserves further investigation.

### Teas (*Camellia sinensis*) and effect of consecutive steepings

The change in elemental concentrations in the consecutive extractions with fresh portions of water on the same material (steepings) was investigated in two kinds of teas – Oolong and Pu‐erh. Traditionally, the preparation procedure for both of these teas involves multiple steepings, and in Pu‐erh the first portion of water is used for rinsing the tea leaves but is not intended for drinking.

**Figure 6:**
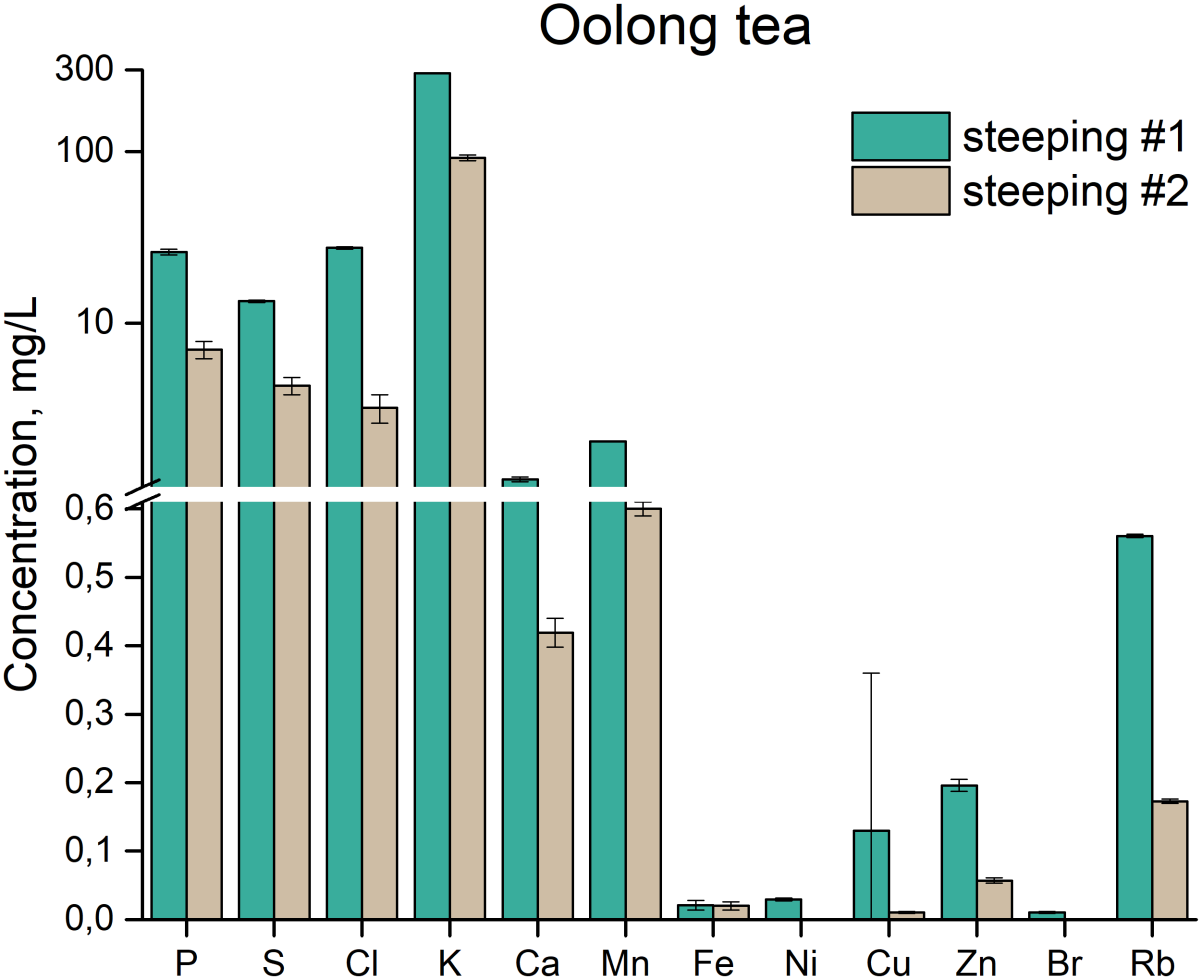
Concentration (mg/L) distribution of the different measured elements for the two steepings of the same leaves for Oolong tea. Each given concentration is an average out of five measurements. Note the logarithmic scale in the range 1,1–300 mg/L.

For the Oolong sample we used two steepings, and the bar diagram on Fig. 6 shows a decrease in concentration in the second steeping for all the elements.

**Figure 7:**
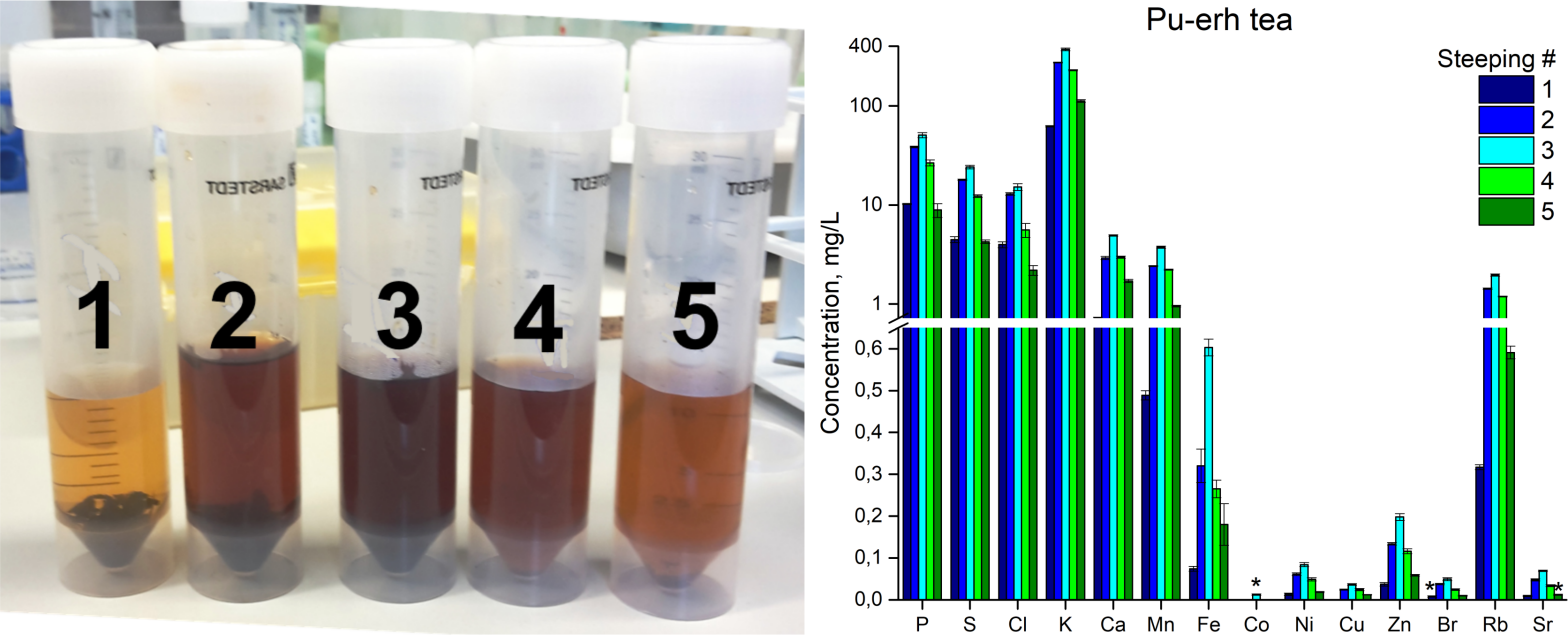
Pu‐erh tea steepings. Left‐hand side: color correlation of the five steepings of Pu‐erh tea. Right‐hand side: concentration (mg/L) distribution of the different measured elements for the five steepings of the same leaves for Pu‐erh tea. Note the logarithmic scale in the range 0.72–400 mg/L. Asterisks mark the elements detected in four out of five measurements.

We expected a similar trend in Pu‐her tea. However, the maximum extraction actually happened at the third steeping for all the elements (Fig. 7, right). This discovery justifies the tradition of pouring boiling water over the same leaves several times. Interestingly, the color of the steepings seems to correlate with the element content, becoming darker and more intense towards the third steeping and decreasing again afterwards (Fig. 7, left).

**Figure 8:**
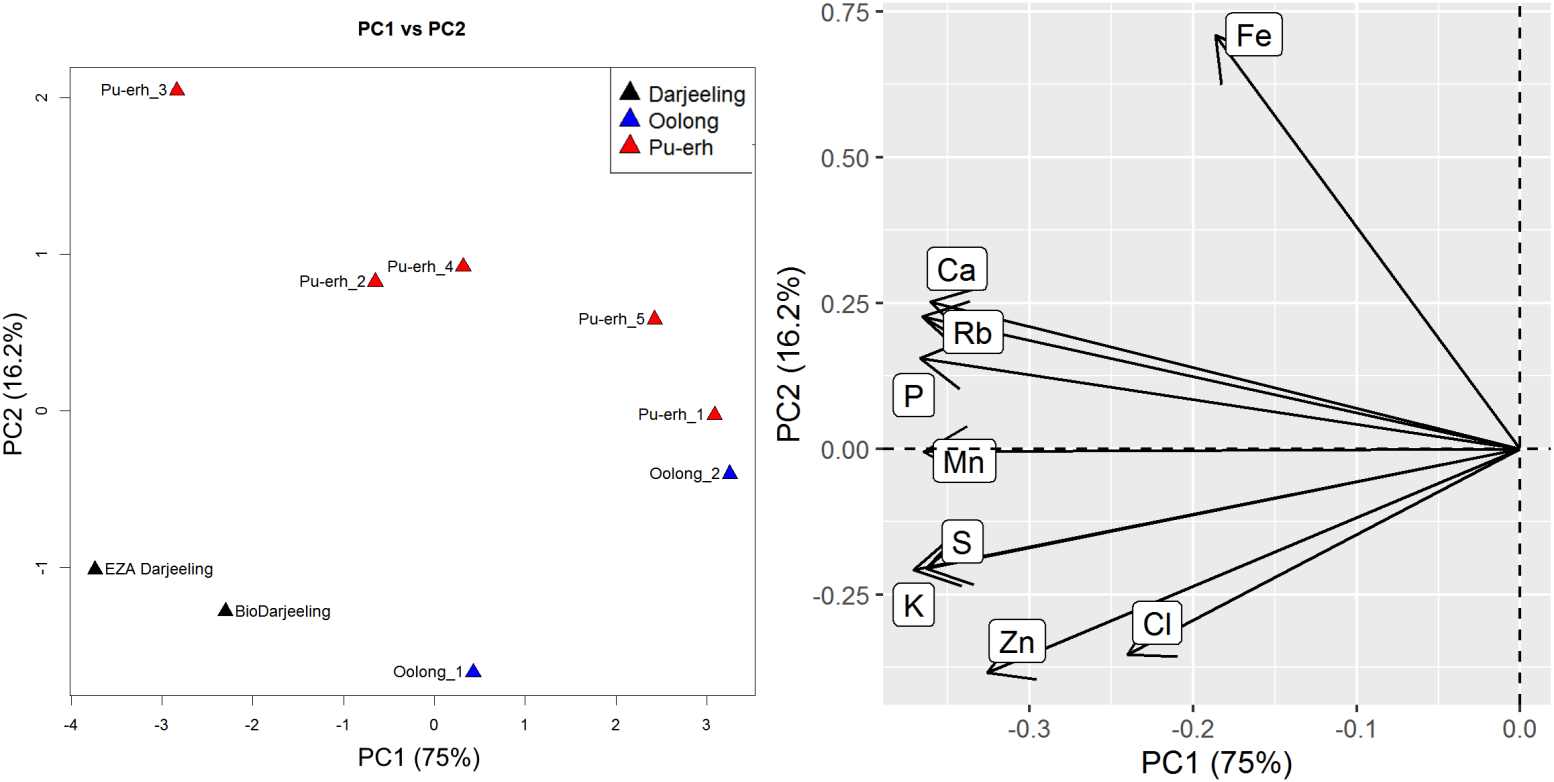
Tea analysis: PCA plot (left) and variables factor map (right).

Finally, we ran a PCA on tea samples exclusively, including all the steepings of Pu‐her and Oolong teas (Fig. 8). In this case the sample pool was rather small; however, similarities in the two Darjeeling samples can be seen, and the steepings of the Pu‐erh tea demonstrate an interesting pattern – pairs of steepings 2 and 4, and 1 and 5 fall close together. At the same time, the two Oolong steepings lie apart from one another.

There are number of papers reporting on successful differentiation and identification depending on the cultivar fingerprint,^27^ the type of the tea^28^ or geographical origin^29^, ^30^, ^31^, ^32^ based on the elemental composition in raw material. We therefore believe that increasing the number of samples that are investigated could provide insights into the traits in the teas that have been prepared as well.

## CONCLUSIONS

The samples of herbal infusions and teas were analyzed with respect to their elemental content using TXRF. This allowed us to quantitatively identify metallic and non‐metallic components, with 13 default elements and three occasional elements. Running PCA analysis aided:

- clear discrimination between teas and herbal infusions;
- differentiation of the Theaceae family (teas), although the Apiaceae, Asteraceae and Lamiaceae families could not be distinguished
- identification of possible influence of packaging when comparing infusions obtained from loose herbal material versus packaged in tea bags
- possible identification of the type of prepared tea.

## Supporting information


**Figure S1.** Spectrum of a clean reflector
**Table S1.** TXRF analysis of the certified reference material NIST 1640 (Trace Elements in Natural Water)
