# Supplementary material for: Total reflection X‐ray fluorescence analysis of elemental composition of herbal infusions and teas
Source: J Sci Food Agric. 2020 May 26;100(11):4226–36. doi: 10.1002/jsfa.10463 (PMC7383995; doi:10.1002/jsfa.10463)
Supplement: Supplementary file 1 — Figure S1. Spectrum of a clean reflector Table S1. TXRF analysis of the certified reference material NIST 1640 (Trace Elements in Natural Water) [file JSFA-100-4226-s001.docx]

Total reflection X-ray fluorescence analysis of elemental composition of herbal infusions and teas

Aleksandra Winkler^a^, Mirjam Rauwolf^a^, Johannes H. Sterba^a^, Peter Wobrauschek^a^, Christina Streli^a^ and Anna Turyanskaya^a^*

^a^ Atominstitut, TU Wien, Stadionallee 2, 1020 Vienna, Austria

^*^ Corresponding author, email: anna.turyanskaya@tuwien.ac.at

The spectrum of the proper clean reflector is shown below (Fig. S1):


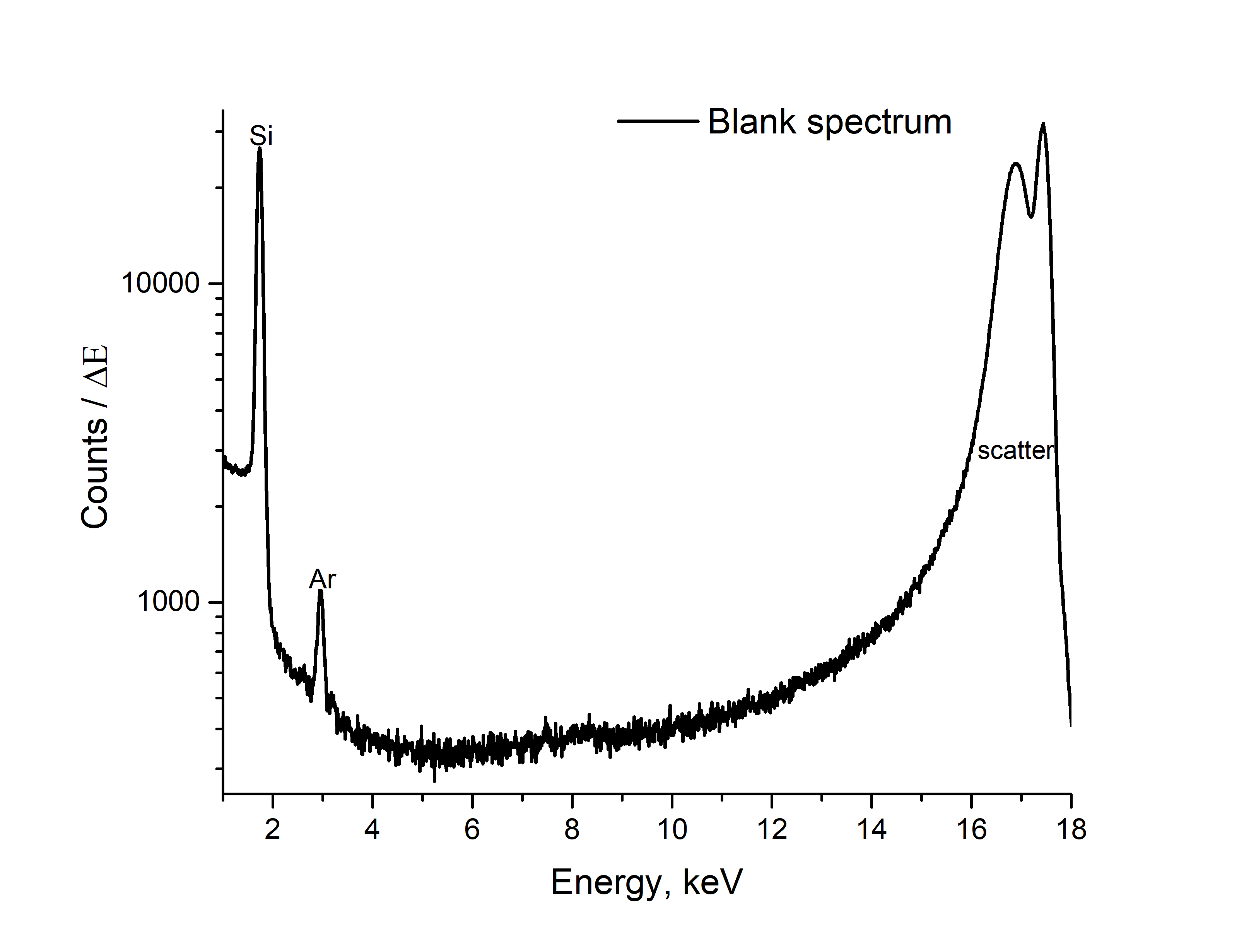


Figure S1. Spectrum of a clean reflector

The validation of the method was performed by measuring the standard reference material NIST 1640 Trace Elements in Natural Water. LOD were evaluated using a measurement time of 1000 s.

Table S1. TXRF analysis of the certified reference material NIST 1640 (Trace Elements in Natural Water)

| **Element** | **Certified value ± uncertainty, µg/L** | **Experimental value ± SD, µg/L** | **LOD, µg/L** |
| --- | --- | --- | --- |
| K | 994 ± 27 | 988.6 ± 83.3 | 51.0 |
| Ca | 7045 ± 89 | 8009.9 ± 317.5 | 29.5 |
| Cr | 38.6 ± 1.6 | 42.6 ± 1.7 | 6.6 |
| Mn | 121.5 ± 1.1 | 136.7 ± 3.5 | 5.8 |
| Fe | 34.3 ± 1.6 | 47.6 ± 4.2 | 1.3 |
| Co | 20.28 ± 0.31 | 23.4 ± 1.5 | - |
| Ni | 27.4 ± 0.8 | 35.0 ± 1.5 | 1.5 |
| Cu | 85.2 ± 1.2 | 98.1 ± 0.9 | 4.0 |
| Zn | 53.2 ± 1.1 | 74.5 ± 1.9 | 5.0 |
| As | 26.67 ± 0.41 | 30.9 ± 2.8 | - |
| Se | 21.96 ± 0.51 | 21.8 ± 2.4 | 0.9 |
| Sr | 124.2 ± 0.7 | 116.4 ± 6.9 | 0.9 |
| Ba* | 148 ± 2.2 | 166.8 ± 28.4 | 51.0 |
| Pb* | 27.89 ± 0.14 | 30.1 ± 3.0 | 2.1 |

*Ba and Pb measured via L-lines

For Co and As LOD could not be calculated due to spectral overlaps.

References:

1. Winkler A. Total reflection X-ray fluorescence analysis of trace elements in black teas and herbal infusions [Internet]. TU Wien; 2017. Available from: http://repositum.tuwien.ac.at/obvutwhs/content/titleinfo/1863692
